# Genipin crosslinking reduced the immunogenicity of xenogeneic decellularized porcine whole-liver matrices through regulation of immune cell proliferation and polarization

**DOI:** 10.1038/srep24779

**Published:** 2016-04-21

**Authors:** Yujia Wang, Ji Bao, Xiujuan Wu, Qiong Wu, Yi Li, Yongjie Zhou, Li Li, Hong Bu

**Affiliations:** 1 Laboratory of Pathology, West China Hospital, Sichuan University, Chengdu, 610041, China; 2 Key Laboratory of Transplant Engineering and Immunology, Ministry of Health, West China Hospital, Sichuan University, Chengdu, 610041, China; 3 Department of General Surgery, The first people’s hospital of Yibin, Yibin, 644000, China; 4 Department of Pathology, West China Hospital, Sichuan University, Chengdu, 610041, China

## Abstract

Decellularized xenogeneic whole-liver matrices are plausible biomedical materials for the bioengineering of liver transplantation. A common method to reduce the inflammatory potential of xenogeneic matrices is crosslinking. Nevertheless, a comprehensive analysis of the immunogenic features of cross-linked decellularized tissue is still lacking. We aimed to reduce the immunogenicity of decellularized porcine whole-liver matrix through crosslinking with glutaraldehyde or genipin, a new natural agent, and investigated the mechanism of the immune-mediated responses. The histologic assessment of the host’s immune reaction activated in response to these scaffolds, as well as the M1/M2 phenotypic polarization profile of macrophages, was studied *in vivo*. The genipin-fixed scaffold elicited a predominantly M2 phenotype response, while the glutaraldehyde-fixed scaffold resulted in disrupted host tissue remodeling and a mixed macrophage polarization profile. The specific subsets of immune cells involved in the responses to the scaffolds were identified *in vitro*. Crosslinking alleviated the host response by reducing the proliferation of lymphocytes and their subsets, accompanied by a decreased release of both Th1 and Th2 cytokines. Therefore, we conclude that the natural genipin crosslinking could lower the immunogenic potential of xenogeneic decellularized whole-liver scaffolds.

It is universally acknowledged that the only definitive treatment for end-stage liver failure is orthotopic transplantation. However, the success rate of liver transplantation is restricted by donor pool shrinkage and recipients’ adverse immune responses to implanted grafts. A much more elegant solution to severe organ failure is tissue engineering, which allows the use of autologous cells from patients to produce a new substitute to replace the damaged organ. The elaborate architecture and complex functions of the liver have defined it as a very challenging solid organ for *ex vivo* regeneration^1^. Searching for an ideal scaffold to mimic the complicated environment within the native liver has become a crucial topic in the field of tissue engineering. In recent years, using a decellularized liver scaffold composed of extracellular matrix (ECM) has been considered a promising strategy^2^,^3^. The decellularized liver ECM maintains the multifaceted composition, three-dimensional ultrastructure, and microenvironmental factors suitable for cell adhesion, differentiation and proliferation^4^.

The methods of decellularization and terminal sterilization of the scaffolds vary widely between different laboratories. All protocols aim to preserve the local environment of the liver ECM as well as to abolish the host tissue responses to the graft following implantation. One way to lower the immunogenicity of implanted grafts is by crosslinking with fixation reagents^5^. Crosslinking can enhance the mechanical strength and reduce the immunogenicity of implanted grafts, which have been successfully applied to organs and tissues such as heart valves^6^, umbilical veins^7^, pericardia^8^, spinal cords^9^ and tracheae^10^. Glutaraldehyde is the most widely used agent for xenograft tissue crosslinking. However, despite the widespread use of glutaraldehyde, problems with calcification and cytotoxicity, as well as the suboptimal growth and durability of glutaraldehyde-treated materials, have been reported^11^,^12^.

In recent years, interest has increased in using genipin, a naturally occurring crosslinking agent, to fix biological materials in order to overcome the disadvantages associated with glutaraldehyde. Genipin was first discovered by Huang *et al.* in 1998 as a substitute for glutaraldehyde^13^. It was demonstrated that the *in vivo* biocompatibility of genipin-treated grafts was better than that of their glutaraldehyde-treated counterparts^14^. However, the effects of these different crosslinking methods on the biocompatibility and host immune response have not been thoroughly investigated, and the fate of crosslinked liver ECM during the tissue remodeling following *in vivo* implantation is largely unknown.

The cytokine secretion profile after exogenous stimulation is a widely studied topic of particular importance in cell-mediated immune responses to xenografts. Two different sets of cytokines are secreted by different subsets of helper T cells (Th1 and Th2) after activation, consequently mediating distinct regulatory and effector functions in the immune response. Meanwhile, functional and chemical polarization of the mononuclear macrophage population was also described for the macrophage phenotype profile^15^,^16^. The pro-inflammatory cytotoxic phenotype, signified as M1, refers to macrophages that secrete large amounts of pro-inflammatory signaling and effector molecules and eliminate intracellular pathogens. The anti-inflammatory phenotype, signified as M2, refers to macrophages that mediate constructive tissue remodeling and promote immune regulation. In the foreign body response to biomaterials, different types of reactions, such as inflammation, migration, proliferation and activation, are evoked by different types of cells aggregated in the implant site^17^. Thus, the ideal scaffolds for liver tissue engineering should direct the desired cell type to migrate to the implant site to ensure hepatocyte survival and proliferation. Therefore, a systematic understanding of the cell phenotype activation to different types of crosslinked ECMs is needed to evaluate the host reaction caused by ECM implants. In our previous research, cell extraction processes employing different detergents were applied to remove the cellular components of a porcine liver and kidney, and these decellularization methods were systematically assessed to obtain an optimal decellularization protocol^18^. In the present study, we further modified the obtained acellular liver scaffolds with glutaraldehyde or genipin. We also performed *in vivo* implantation of crosslinked and un-crosslinked decellularized porcine livers in a rat abdominal wall repair model for 21 days to assess their reconstruction ability through investigation of macrophage polarization. To illuminate the mechanism of distinct immune cell polarization triggered by different liver materials, the human immune responses against these scaffolds were analyzed, and the Th1/Th2 cytokine profiles were determined by measuring the secreted cytokines of human peripheral blood mononuclear cells (PBMCs). The objectives of this study were to evaluate the modification of the decellularized livers by glutaraldehyde or genipin and to assess the matrices’ potential interactions with immune cells and the local environments of recipients.

## Results

### Characterization of the decellularized liver allografts

**Figure 1:**
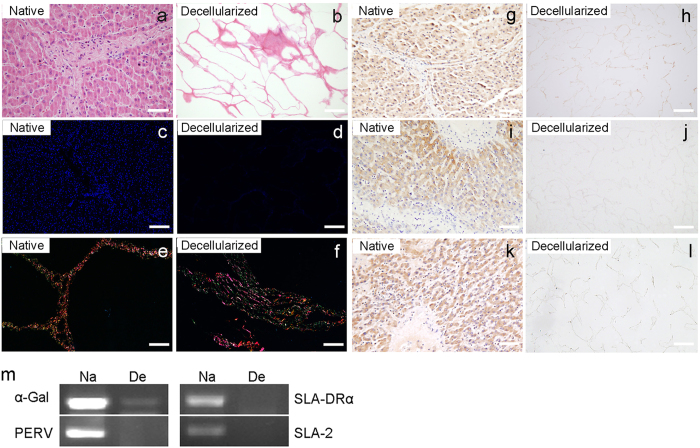
Analysis of decellularized liver materials. (**a**,**b**) Representative H&E staining of the ultrastructure of native liver and decellularized liver matrix. Scale bars = 100 μm. DAPI staining (**c**,**d**) and Sirius Red staining (**e**,**f**) illustrate a lack of residual cellular components and the preservation of matrix components in the decellularized ECM. Scale bars = 200 μm. Immunohistochemical staining of native and decellularized liver tissues for α-Gal (**g**,**h**), SLA-2 (**i**,**j**), SLA-DRα (**k**,**l**) are shown. Scale bars = 100 μm. (**m**) Semi-quantitative PCR for the α-Gal, SLA-DRα, PERV, and SLA-2 antigens in the liver scaffolds. Na = native liver; De = decellularized liver matrix.

As shown in Fig. 1a, the decellularized livers appeared to be free of cell components, and the ultrastructure of the liver ECM was retained. The removal of the nucleus remnants was further confirmed by DAPI staining (Fig. 1c,d), indicating satisfactory decellularization. Sirius Red staining for different types of collagen and proteoglycan indicated an overall retention of ECM components after decellularization (Fig. 1e,f). To verify the decellularization uniformity, ECM samples were taken from 12 different areas (A1 to A12) as shown in Supplementary Fig. S1a, including the right, median, left and caudate lobes of the liver, as well as from the porta hepatis to the lobe margin. The DNA extract was subjected to agarose gel electrophoresis to demonstrate complete removal of DNA fragments (Supplementary Fig. S1b). Hematoxylin and eosin (H&E) staining also indicated that the present decellularization protocol could eliminate the cellular components completely regardless of the areas of the porcine liver (Supplementary Fig. S1c–n). According to previously reported investigations, the Gal epitope in the ECM is a strong immune inducer that may cause hyperacute rejection in the clinic^19^. In the present study, the presence of α-Gal was compared between native porcine livers and ECM scaffolds by immunohistochemistry. The results showed that there was a significant reduction in the α-Gal epitope in the decellularized liver matrix (Fig. 1g,h, Supplementary Fig. S2a–c). Immunochemistry assays of swine leukocyte antigen 2 (SLA-2) and swine leukocyte antigen DRα (SLA-DRα) were carried out in native liver and ECM scaffolds to show the removal of these antigens which can adversely affect transplantation (Fig. 1i,l, Supplementary Fig. S2d–i). Additionally, the presence of potential immunogenic or pathogenic antigen genes was investigated by PCR (Fig. 1m). The absence of α-Gal, SLA-DRα, PERV, and SLA-2 DNA sequences in the decellularized matrix provided a solid argument for the elimination of xenogenic antigens and pathogen genes.

### Crosslinking characteristics

**Figure 2:**
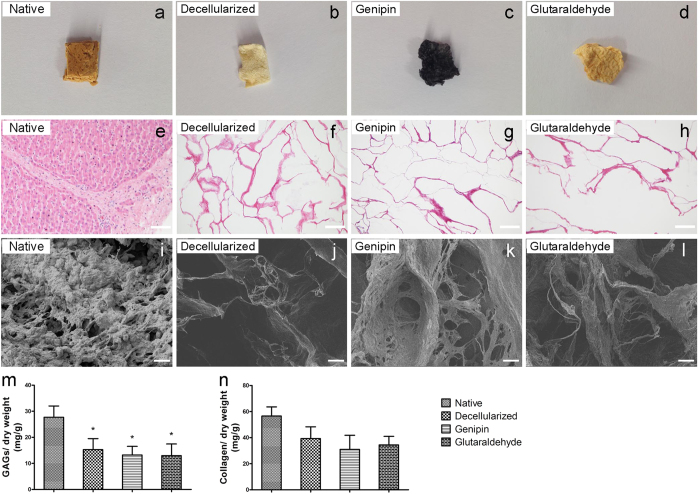
Morphological and quantitative analysis of liver materials. (**a**–**d**) Macroscopic appearance of native liver, decellularized liver ECM, genipin-fixed ECM and glutaraldehyde-fixed ECM. (**e**–**h**) Histological images of liver samples stained with H&E after dehydration and sterilization processes. Scale bars = 100 μm. (**i**–**l**) Scanning electron microscopy of the liver materials. Scale bars = 20 μm. (**m**) The concentration of GAGs in the liver materials. (**n**) The concentration of collagen in the liver materials. The collagen and GAG contents were normalized to the initial dry weight of the sample. *p < 0.05 with respect to the native liver group. All data are given as the mean ± SEM.

The crosslinking reagents that were used on the decellularized porcine livers in this research included glutaraldehyde and genipin (Fig. 2a–d). Figure 2e–h shows histological images of native liver, decellularized liver ECM, genipin-fixed ECM, and glutaraldehyde-fixed ECM stained with H&E, indicating that fixation did not change the appearance of the liver ECM. Scanning electron microscopy results showed that the decellularized matrix was composed of fibrils and sheets of matrix components and that the genipin- or glutaraldehyde-fixed tissues were characterized with dense extracellular fibrils and more porous surfaces compared with un-fixed decellularized matrix (Fig. 2i–l).

We further quantified the glycosaminoglycans (GAGs) and total collagen to analyze the maintenance of ECM components after crosslinking. Although there was a reduction of GAGs in the decellularized matrices compared to the native liver, the GAG content failed to achieve statistical significance among the decellularized liver ECM, genipin-fixed ECM and glutaraldehyde-fixed ECM (Fig. 2m). Similarly, a colorimetric collagen quantification assay showed that both crosslinking methods preserved most of the collagen in the ECM (Fig. 2n). Meanwhile, the presence of specific ECM molecules, including collagen I and fibronectin, was confirmed by immunohistochemistry (Supplementary Fig. S3). In general, the distributions of these ECM components were similar in the decellularized scaffold matrices and crosslinked matrices. The levels of several growth factors that are important for liver angiogenesis and regeneration were also evaluated in the native liver and liver scaffolds, such as HGF, VEGF, bFGF, and IGF (Supplementary Fig. S4). The immunochemistry results indicate that at least parts of each growth factor were maintained in the crosslinked matrices. Therefore, taken together, these data demonstrate that the ECM structure and components were well retained after crosslinking with glutaraldehyde or genipin.

### Biocompatibility of liver ECM

**Figure 3:**
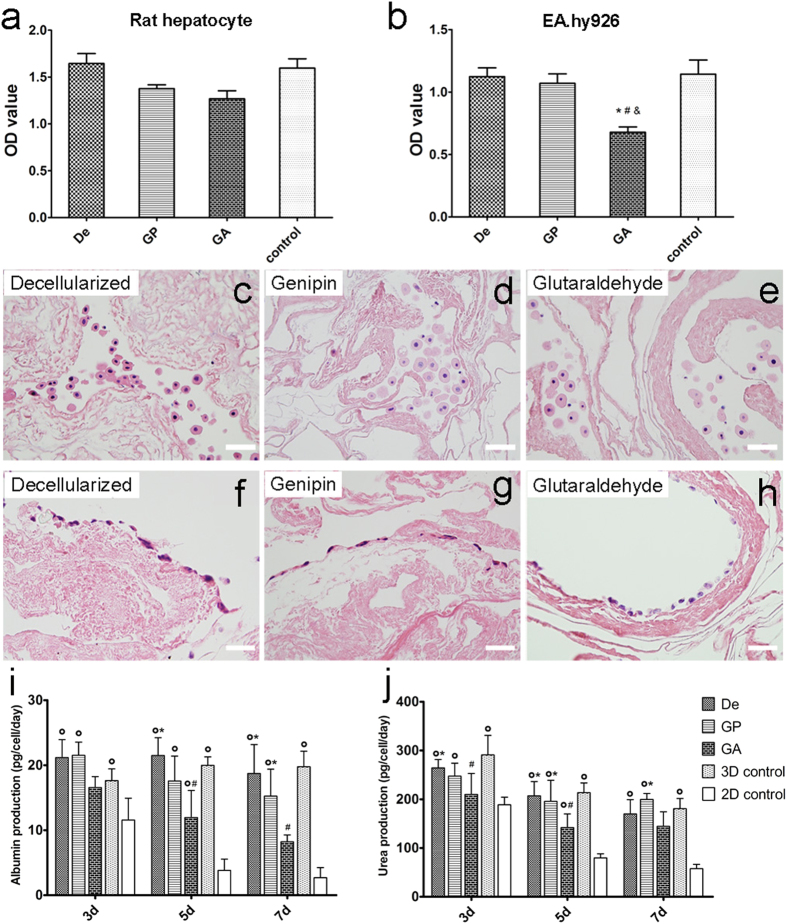
Cells seeded on the uncrosslinked and crosslinked liver ECMs. (**a**) Cell viability assay for rat primary hepatocytes seeded on liver ECMs at day 3. (**b**) The effect of liver ECMs on the cell viability of EA.hy926 endothelial cells at day 3. The normal single layer culture was used as control. (**c**–**e**) H&E staining of liver ECMs with rat primary hepatocytes. Scale bars = 50 μm. (**f**–**h**) H&E staining of human of liver matrices with EA.hy926 endothelial cells. Scale bars = 50 μm. (**i**) Albumin production of rat primary hepatocytes in each group at day 3, day 5 and day 7. (**j**) Urea production of rat primary hepatocytes at day 3, day 5 and day 7. De = decellularized; GP = genipin; GA = glutaraldehyde; 3D control = sandwich culture; 2D control = single layer culture. *p < 0.05 with respect to GA group, ^#^p < 0.05 with respect to 3D control group, ^o^p < 0.05 with respect to 2D control group. All data are given as the mean ± SEM (n = 6/each group).

To determine whether crosslinked matrices may be cytotoxic to cells and to reveal the capacity of ECMs to maintain cell viability, rat primary hepatocytes and the human EA.hy926 endothelial cell line were cultured, respectively, with decellularized, genipin-fixed and glutaraldehyde-crosslinked matrices for 3 days. As shown in Fig. 3a,b, genipin crosslinking did not appear to be significantly suppressive with regards to the viability of primary hepatocytes and endothelial cells, which are the most important cells for liver function. And glutaraldehyde crosslinking caused a reduction of EA.hy926 endothelial cell number after 3 days (P < 0.05). H&E staining revealed that hepatocytes had aggregated into clusters in the ECM scaffold (Fig. 3c–h). No distinctions were found among the three groups with regard to hepatocytes, whereas the morphology of endothelial cells cultured on the glutaraldehyde-crosslinked matrix were different from cells cultured on others. Cells were observed tightly attached to the surface of the ECM and exhibited a typically flat morphology in the genipin and decellularized groups. In contrast, endothelial cells in the glutaraldehyde group were scattered and formed into a loose attached pattern on the matrix. Additionally, during 7 days of *in vitro* culture, hepatocyte function was generally maintained when cultured with genipin crosslinked liver ECM. While hepatocyte albumin production was obviously lower in the glutaraldehyde-crosslinking group than that in decellularized group and the 3D control group after day 5, the levels of urea production in the rat hepatocyte culture in the decellularized and genipin groups were similar to that in the 3D control group (Fig. 3i,j). All the results indicate that glutaraldehyde crosslinking may have a stronger cytotoxic impact than genipin treatment.

### Characteristics of implanted liver material *in vivo*

**Figure 4:**
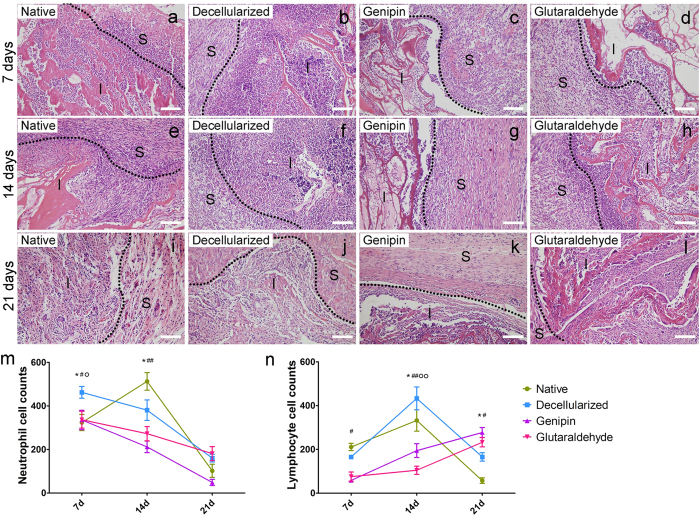
Histologic analysis of the liver xenografts. (**a**–**l**) The host immune responses towards native liver, decellularized liver ECM, genipin-fixed ECM, and glutaraldehyde-fixed ECM at 7 days, 14 days, and 21 days post-surgery are shown. Scale bars = 100 μm. The dotted line indicates the border of the implants and surrounding tissue. Abbreviations: S = surrounding tissue; I = implanted porcine liver materials. (**m**,**n**) Quantification of neutrophil and lymphocyte infiltration. The numbers of cells were counted in at least five different areas for each slide. *p < 0.05 with respect to the native group compared to the decellularized group; ^#^p < 0.05 with respect to the genipin group compared to the decellularized group; ^##^p < 0.01 with respect to the genipin group compared to the decellularized group; ^o^p < 0.05 with respect to the glutaraldehyde group compared to the decellularized group; ^oo^p < 0.01 with respect to the glutaraldehyde group compared to the decellularized group. All data are given as the mean ± SEM (n = 3/each group).

To evaluate the host immune response and biocompatibility of xenotransplantation, we implanted the liver material in the rat partial-thickness abdominal wall defect model. The liver materials were divided into four groups: native liver, decellularized matrix, genipin-fixed decellularized matrix, and glutaraldehyde-fixed decellularized matrix. Macroscopically, both the native and decellularized materials became smaller over time and could hardly be observed 21 days post-implantation. In contrast, the implanted genipin and glutaraldehyde groups showed little signs of degradation of the implanted scaffolds. H&E staining revealed the significant differences in inflammation among the four groups (Fig. 4a–l). A significantly greater degree of lymphocyte infiltration was observed in the native and decellularized groups. The genipin group presented a relatively mild degree of lymphocyte and neutrophil infiltration (Fig. 4m,n). However, the glutaraldehyde group showed higher degrees of inflammation compared to the genipin group. At 21 days post-implantation, the inflammatory reactions of the native and decellularized groups were significantly reduced because the implants had been degraded and could hardly be observed in the implant site. At this time point, the immunochemistry towards vascular cell marker CD31 and fibroblast marker vimentin also revealed that angiogenesis and the repopulation of fibroblast-like cells were more robust in the genipin group (Supplementary Fig. S5).

**Figure 5:**
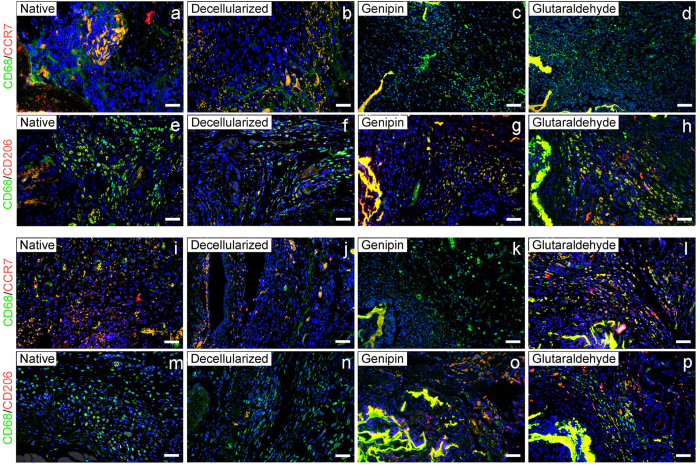
M1/M2 macrophage phenotype distribution in the host immuneresponses to the porcine liver matrices at 7 days and 21 days post-surgery. Representative immunofluorescent images presenting host macrophage polarization responses to liver matrix materials 7 days (**a**–**h**) and 21 days (**i**–**p**) post-implantation. M1 cells were co-stained with CCR7 and CD68, and M2 cells were co-stained with CD206 and CD68. Scale bars = 100 μm. CCR7/CD206 = red; CD68 = green; DAPI = blue.

The macrophage phenotype was characterized by immunofluorescence methods at the 7 day and 21 day time points, which represented the early stage and later stage of host responses to the implantation, respectively. Macrophages presented in all four groups of liver materials, with most of the macrophages detected on the external surface of the implant, although there were obviously distinct differences in the distribution of the M1 and M2 phenotypes. After 7 days of implantation (Fig. 5a–h), the native group showed the most intense M1 macrophage responses compared to the other three groups. The M2 macrophage distribution was opposite from the M1 phenotype. The immunofluorescence showed larger numbers of CD68+CD206+ cells present in the cross-linked groups than that present in the native and decelluarlized groups. By 21 days post-implantation (Fig. 5i–p), the proportion of M1/M2 surrounding the implant of the genipin group remained the same as on day 7, while the glutaraldehyde group was characterized by a shift from an M2-dominant phenotype to a mixed M1/M2 phenotype by the later period. This indicates that both a constructive and destructive tissue formation were present in the host immune responses induced by the glutaraldehyde-treated implant 21 days post-surgery.

### Carboxyfluorescein succinimidyl ester (CFSE)-based assay of human PBMC proliferation

**Figure 6:**
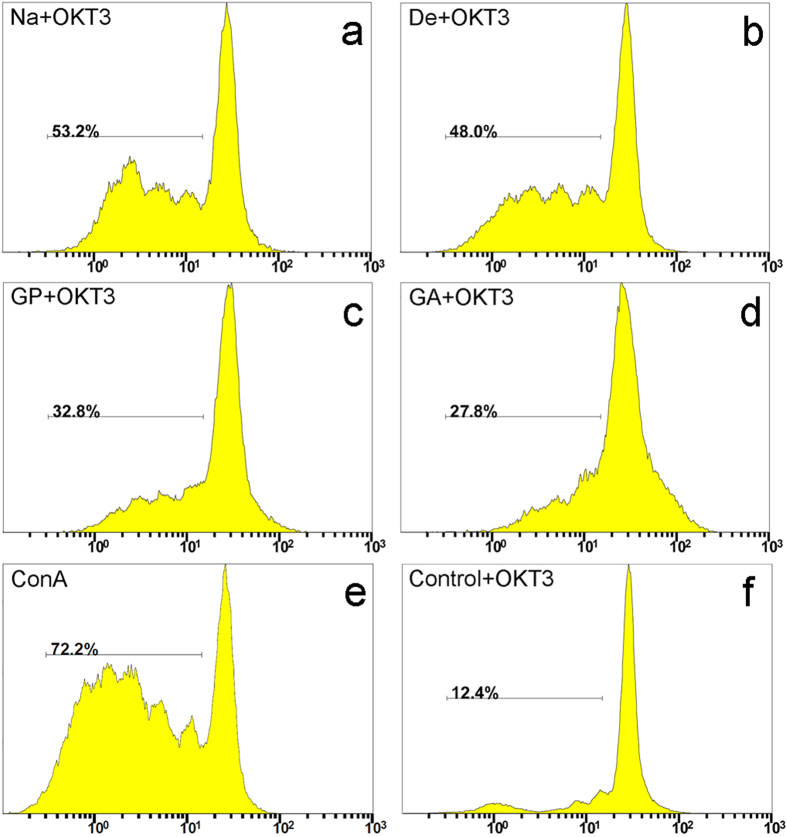
Proliferation properties of PBMCs in co-culture with protein extracts in the CFSE-labeling assay. (**a**–**f**) Representative FACS histograms of immune cells co-cultured in the presence of a low-dose OKT3 stimulus. Proliferation responses without co-culture with protein extracts were used as a negative control. Con A served as a positive control. Na = native liver; De = decellularized; GP = genipin; GA = glutaraldehyde. The depicted line defines the level of proliferated immune cells.

To further analyze the mechanism of immune cell activation and polarization triggered by liver ECM, the CFSE labeling assay was used to track the division of human immune cells. We first analyzed the protein extracts in co-cultures with human PBMCs regarding the extracts’ ability to induce cell proliferation (Supplementary Fig. S6). Distinctions were observed between the proliferation level of the Na group and the levels of the other three groups. To fully understand the possible immune responses triggered by the fixed matrices, we assessed the results of using the protein extracts as a stimulus secondary to low-dose OKT3, the anti-CD3 monoclonal antibody, an immune cell stimulus. Representative histograms from flow cytometry are shown in Fig. 6. After application of low-dose OKT3 stimulation, the cultures without any extraneous protein, serving as a negative control, achieved a PBMC proliferation level of 12.4%, confirming the reliability and suitability of OKT3 stimulation. As expected, co-cultures in the native group reached a higher proliferation level than did those in the decellularized, genipin, and glutaraldehyde groups. More interestingly, the genipin and glutaraldehyde groups were identified as having less negative effects on PBMC proliferation compared with the decellularized group.

### Effects of liver materials on immune cell subpopulation proliferation

**Figure 7:**
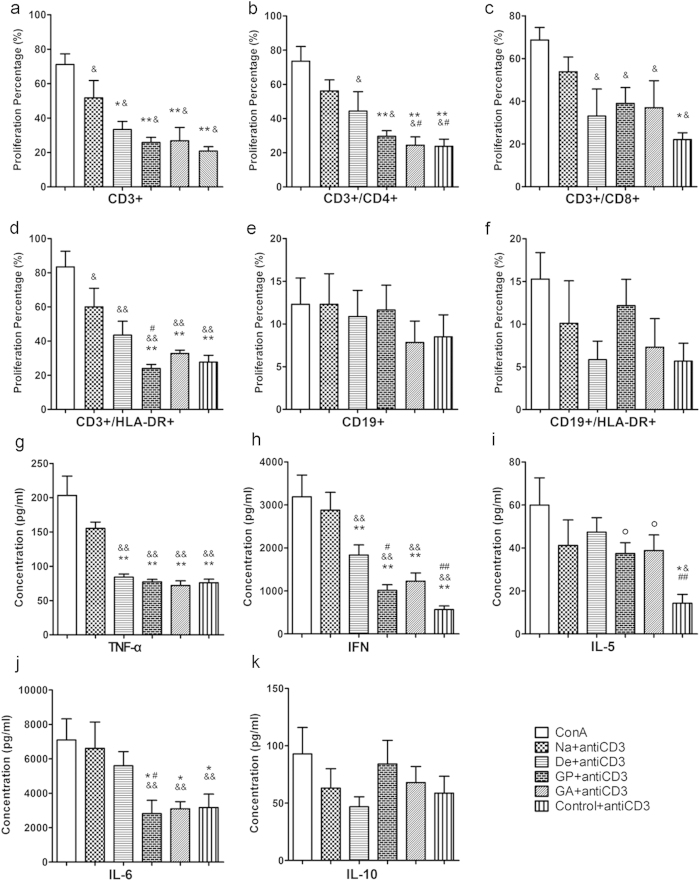
Impact of porcine liver matrices on T cell and B cell subpopulation proliferation and Th1/Th2 cytokine secretion in co-cultures in the presence of OKT3. (**a**–**f**) The proliferation patterns of T cells, B cells and their subsets were analyzed using anti-human CD3, CD8, CD4, HLA-DR and CD19 antibodies. (**g**–**k**) Th1 and Th2 cytokine levels for TNF-α, IFN-γ, IL-5, IL-6 and IL-10 are shown for co-cultures of PBMCs alone or in combination with protein extracts of the porcine matrix. Negative controls without any proteins from the liver materials in OKT3-coated wells are included. Na = native liver; De = decellularized; GP = genipin; GA = glutaraldehyde. *p < 0.05 with respect to the native group; **p < 0.01 with respect to the native group; ^#^p < 0.05 with respect to the decellularized group; ^##^p < 0.01 with respect to the decellularized group; ^o^p < 0.05 with respect to the control group. All data are given as the mean ± SEM.

The degree of proliferation of the T cell and B cell subpopulations with or without OKT3 stimulation was also analyzed (Fig. 7a–f and Supplementary Fig. S7). Interestingly, after OKT3 treatment, native +OKT3 exhibited intense and significant induction of CD3+ cell proliferation, whereas decelluarlized +OKT3 showed a decrease in the same proliferation index, which was nearly abrogated in genipin +OKT3 and glutaraldehyde +OKT3 compared with the negative control +OKT3. Similarly, we observed an intriguing distinction among the different groups with regards to the CD3+CD8+ lymphocytes. Low proliferation levels were detected for decellularized +OKT3, genipin +OKT3 and glutaraldehyde +OKT3 in co-cultures. The proliferation response of CD3+CD4+ lymphocytes showed the most intense level in native +OKT3, and decellularized +OKT3 induced higher proliferation levels compared with the level in the crosslinking and control groups. We also examined the proliferation profile of a T-cell subset that can express human leukocyte antigen DR (HLA-DR), which is an important marker of activated human immune cells. Significantly lower proliferation values were obtained for genipin +OKT3 compared with glutaraldehyde +OKT3, decellularized +OKT3 and native +OKT3. In contrast to the results obtained for CD3+ cells, the proliferation pattern of CD19+ lymphocytes was maintained at a low level, regardless of the groups analyzed. It is worth mentioning that IgG and IgM production, which is the crucial index of B-cell activation, were also examined in all groups (Supplementary Fig. S8). There were no detectable differences in IgG or IgM levels with respect to the control group. It could be demonstrated that in contrast to the CD3+ T cells and related CD3+ subsets in their proliferation characteristics, CD19+ B cells can barely be effectively induced by OKT3 or liver tissue extracts.

### Cytokine profiles elicited by co-culture with ECM protein extracts

The cytokine profiles of human PBMCs incubated with protein extracts and without supplemental suboptimal OKT3 stimulation were measured (Supplementary Fig. S9). The levels of interleukin-2 (IL-2), interleukin-4 (IL-4), interleukin-5 (IL-5), interleukin-6 (IL-6), interleukin-10 (IL-10), tumor necrosis factor-α (TNF-α), and interferon-γ (IFN-γ), which are important components of the Th1/Th2 balance, were all maintained at relatively low levels. We also analyzed the cytokine release pattern of human PBMCs co-cultured with porcine liver tissues in the presence of OKT3 stimulation (Fig. 7g–k). Broadly speaking, higher concentrations of all of the examined cytokines were detected after OKT3 treatment. In terms of Th1 cytokine induction by PBMCs, co-cultures in the native +OKT3 group elicited higher amounts of TNF-α than other co-cultures. Additionally, stimulation in the native +OKT3 group triggered much higher IFN-γ secretion than that in the decellularized and fixed groups. There was also a significant difference between the decelluarlized +OKT3 group and the genipin +OKT3 group (p < 0.05). In the Th2 cytokine profile analysis, no significant differences were observed in the protein levels of IL-5 and IL-10 among the four groups, and the release of these two cytokines was nearly negligible (<84.21 ± 20.55 pg/mL). However, the IL-6 levels in the native and decellularized groups were much higher than in the genipin and glutaraldehyde groups after 5 days of incubation.

### Cytokine profiles of implants *in vivo*

**Figure 8:**
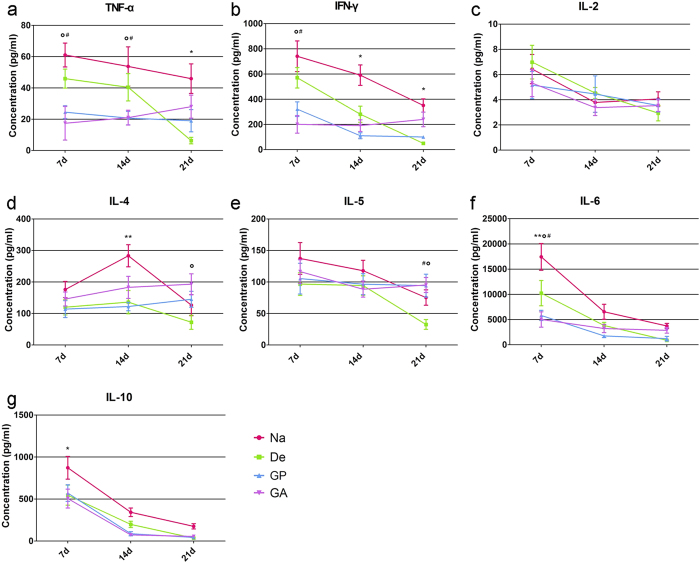
Th1/Th2 cytokine secretion profiles of material implants *in vivo*. (**a**–**g**) Levels of TNF-α, IFN-γ, IL-2, IL-4, IL-5, IL-6 and IL-10 in abdominal wall implantsare shown to indicate the Th1/Th2 profile of each group. Na = native liver; De = decellularized; GP = genipin; GA = glutaraldehyde. *p < 0.05Na group with respect to the decellularized group; **p < 0.01 native group with respect to the decellularized group; ^#^p < 0.05 genipin group with respect to the decellularized group; ^o^p < 0.05 glutaraldehyde group with respect to the decellularized group. All data are given as the mean ± SEM.

Because we observed a cytokine polarization shift in the PBMC proliferation assay *in vitro*, Th1/Th2 polarization in abdominal wall implants at 7 days, 14 days and 21 days were also detected (Fig. 8). In general, all cytokines tested were above the limit of detection. Consistent with the cytokine profile of PBMCs in the presence of OKT3 stimulation, Th1 cytokine TNF-α and IFN-γ were significantly reduced after crosslinking with genipin or glutaraldehyde at day 7 and day 14 (compared to the decellularized group, p < 0.05 for TNF-α level at day 7 and day 14, p < 0.05 for IFN-γ level at day 7). There were no significant differences in the concentrations of IL-2. Moreover, in terms of Th2 cytokines, the IL-4 concentration in the genipin and glutaraldehyde implants increased over time and reached the highest level at day 21. And both the IL-4 and IL-5 levels in the decellularized groups were all maintained at rather low levels, which may be attributed to the rapid degradation of the decellularized materials. The distinctions of the IL-6 secretions among the four groups were the most significant at the early stage of implantation, because genipin and glutaraldehyde triggered lower levels of IL-6 (p < 0.05, compared with the De group at day 7). For IL-10 secretion, a significant difference was found only between the native and decellularized groups at day 7 (p < 0.05). It is worth mentioning that TNF-α and IFN-γ, which are two crucial pro-inflammatory cytokines that facilitate the cellular immunity response, had higher levels in the native and decellularized groups throughout the host response, in accordance with the H&E staining results.

## Discussion

In our previous study of liver tissue engineering, we presented an efficient decellularization protocol suitable for a human-sized whole liver and kidney and discussed the use of different chemical detergents. The present study is one of the first to characterize the appearance of acellular porcine liver fixed with genipin or glutaraldehyde, describe the effects of these two agents on the decellularized matrix with regards to immunogenic properties and discuss the mechanism involved in the relationship between matrix crosslinking and the biocompatibility of host tissue. Our experiment suggests that a novel crosslinked acellular liver scaffold with reduced immune induction offers a source of engineered tissue for functional liver reconstruction.

The decellularization and sterilization methods used during the ECM manufacturing process can markedly affect the immunogenicity and functional outcome of the material^2^. Unfortunately, despite the known anti-rejection characteristic of the decellularized matrix, xenogenic materials planted into human patients or recipient animals causes mixed results^20^,^21^. Rieder *et al.* reported attenuated lymphocyte and monocyte migration after vascular tissue decellularization^22^. Meanwhile, recent research has reported that no increased immune response was observed with either allogeneic or xenogeneic liver decellularized subcutaneous transplants by counting peripheral bloodcells^23^. Consistent with that finding, our results showed no significant differences in blood counting among the four groups of liver materials after implantation (Supplementary Fig. S10). However, immune responses induced by decellularized porcine matrices were still present in the implanted site. In addition, the inconsistencies can also be attributed to different experimental design parameters, such as the decellularization and sterilization protocols, graft implantation site, and measurement methods used to characterize the immune reactions.

Glutaraldehyde can react with the specific amino groups of lysine residues in proteins, forming a covalent bond that generates inter-chain crosslinking^24^. This crosslinking stabilizes the ECM against chemical and enzymatic degradation and, most importantly, alters the intrinsic immunogenicity of the xenogenic matrix. The preparation of glutaraldehyde-crosslinked grafts, such as heart valves, has been put into clinical practice for a long time^25^. However, disadvantages of glutaraldehyde fixation have been reported, such as dystrophic calcification^26^, pro-inflammatory effects on macrophage-like cells^27^ and a cytotoxic effect that may impair the *in vivo* biocompatibility of fixed tissues^28^. In the work here, the glutaraldehyde-treated liver matrix showed disrupted host tissue events in abdominal wall repair models, and mixed macrophage polarization profiles that involved both M1 and M2 phenotypes. In addition, the immunogenicity of glutaraldehyde-crosslinked ecm was not eliminated, as assayed by proliferation of human pbmcs co-culture *in vitro*. Genipin crosslinking of acellular grafts has been proven to provide tissue stabilization and reduce host immune reactions, but with much lower cytotoxicity, which is associated with a significant decrease in the release of untreated or depolymerized reagent. This may contribute to the lower cytotoxicity of the genipin crosslinking. Furthermore, it is quite interesting that through immunochemistry analysis of liver ECM implants, we discovered that angiogenesis was present in the genipin-crosslinked group. Small vessels (CD31+) were observed inside the implantation site of the genipin-treated scaffold, indicating new vessel formation post-implantation, whereas there were few CD31+ endothelial cells inside the implanted ECM in the other groups. Meanwhile, immunochemistry of the fibroblast marker vimentin also demonstrated that fibroblast cells migrated in the genipin-crosslinked ECM. These results suggest the potential ability of genipin crosslinking to promote beneficial tissue repair because both angiogenesis and fibroblast migration are important prerequisites for the long-term survival of implanted cells and tissues^29^,^30^.

The present study was conducted to evaluate the immune events in response to un-crosslinked and crosslinked liver matrices in a rat model of abdominal wall muscle repair. Interestingly, the host responses involved a large amount of mononuclear cell recruitment, a sign conventionally associated with inflammation; this could be observed in all four groups of liver materials, albeit to different degrees. However, crosslinking with genipin enabled the liver scaffold to shift the macrophage polarization profile to a predominantly M2 phenotype through 21 days post-implantation. According to Brown and his colleagues^31^, increases in the M2 macrophage phenotype and a higher ratio of M2/M1 within the implant site were associated with a more favorable and beneficial histologic prognosis of tissue repair. Moreover, it is clear that the glutaraldehyde-crosslinked decellularized liver scaffold induced a more pro-inflammatory reaction with more intense M1 macrophage infiltration in the implant site than did the genipin-crosslinked scaffold. These results agree very well with the previous study reporting that glutaraldehyde may have rendered the decellularized scaffold surface resistant to cellular binding and induced cytotoxocity by reducing cellular attachment, killing attached cells, and altering the behavior of surviving cells^27^.

The components of the ECM may serve as a stimulatory signal that evokes host cellular and humoral immune responses^32^,^33^. The distinction among liver materials with regards to the ability to invoke host responses in our *in vivo* study led us to further ascertain the specific chemical and physical immune responses induced by xenogenic ECM. An important function of the inflammatory response is the proliferation and recruitment of monocytes towards immunological insult^34^. In the present study, PBMC proliferation was activated by the anti-CD3 antibody OKT3, which was first described by Matsuyama *et al.*^35^. Using this approach, we demonstrated a reduced but not yet abolished monocyte proliferation in the decellularized liver. The higher proliferation level of PBMCs that had been co-cultured with glutaraldehyde-treated matrix protein is consistent with the local observation in our *in vivo* studies, suggesting a cytotoxic influence of glutaraldehyde on the liver ECM. More importantly, the specific lymphocyte subsets activated by ECM were identified by flow cytometry. Previous reports including the use of gene-knockout mice^36^ have shown that CD4+ T cells, but not CD8+ cells, are strongly involved in initiating xenogenic transplantation rejection. In line with these previous studies, in the current research, CD3+CD8+ cell proliferation showed no significant difference among the decellularized group and crosslinked groups. Meanwhile, a higher percentage of proliferating CD3+CD4+ T cells were detected in the native and decellularized groups, suggesting potential T helper cell activation. As a consequence, we suggest that crosslinking might reduce inflammatory responses mainly by inhibiting CD3+CD4+ cell proliferation.

Specifically considering the activated cell subpopulations, we identified higher percentages of proliferated CD3+HLA-DR+ cells in the native and decellularized liver matrix groups. Activation of lymphocytes can be influenced by many inflammatory mediators released in response to xenograft interaction, such as cytokines and chemokines, thus determining the type and strength of the host immune responses. During the foreign body response of biomaterials, secretion of pro-inflammatory cytokines such as IL-6 and TNFα can recruit additional macrophages to the implantation site and lead to degradation^37^. In our comparative analysis of cytokine secretion profiles *in vitro*, both Th1 and Th2 cytokines were found to be involved in the induction of immune cell proliferation in response to liver tissue protein stimulation. Nevertheless, Allman *et al.* described porcine ECM as eliciting an immune response that is predominately Th2-like, consistent with a remodeling reaction, rather than rejection^38^. It has also been shown that Th2 cytokines play a dominant role in the host responses caused by xenogenic small-intestinal submucosal matrix implantation, whereas Th1 secretion was relatively low^39^. These conflicting results with respect to our obtained data might be explained by the distinct and specific characteristics of the porcine small intestine compared with the liver, which features abundant cellular and extracellular proteins and complex three-dimensional structures. The observation reported herein regarding the different results of Th1/Th2 polarization *in vitro* and the M1/M2 polarization *in vivo* were possible because of the simplified *in vitro* system employed as a model of the more complicated host immune responses. However, the *in vitro* results still provide a clue for a comprehensive understanding of the lymphocyte and macrophage participation in a foreign body response.

The longer duration of genipin-crosslinked liver matrix implant compared with non-crosslinked matrix was observed *in vivo* in the present study, which was a result of introduction of intermicrofibrillar crosslinks between adjacent collagen microfibrils induces by genipin^40^. The crosslinking process not only physically hinders the penetration of enzymes into liver scaffolds, but also prevents the infiltration of host immune cells. This allows the liver ECM obtained a stronger ability to withstand degradation forces experienced *in vivo*. However, although enhanced stability is a desired feature of tissue engineering scaffolds, biomaterials that degraded too slowly are associated with presence of adverse innate immune responses, such as chronic inflammation, accumulation of dense fibrous tissue, and grafts failure^41^. Therefore, a suitable tissue remolding needs an equilibrium between host tissue formation and implanted matrix degradation. Previous works have shown that biocompatibility and degradation of crosslinked biomaterials can be achieved by altering the concentration of genipin^42^,^43^. Thus, careful selection is required of the protocol optimization with regards to the genipin concentration and incubation time. Comparable analyses of other aspects of crosslinking, such as mechanical properties, calcification and scaffold degradation, have not yet been systemically performed. Although the beneficial aspects of crosslinking are covered in the present study, this technique also causes some controversial issues in the bioengineering of scaffolds, such as partial denaturation and matrix turnover. All these concerns require further analysis. Therefore, our future studies will include the investigation of those aspects as well as the development of a clinical approach to avoid undesirable immune reactions by utilizing the crosslinking agents.

## Materials and Methods

### Organ preparation

All experiments involving pigs and rats were performed in accordance with committee regulations approved by the Animal Experiment Center of Sichuan University. Livers were harvested from 15–20 kg male Bama miniature pigs (Guangxi, China) for use as scaffold sources. For native liver, decellularized ECM, genipin-crosslinked ECM, and glutaraldehyde-crosslinked ECM, 3 pigs were used for each group. The pigs were anesthetized with ketamine (6 mg/kg body weight; Kelun, Chengdu, China) and xylazine (10 mg/kg; Kelun, Chengdu, China) through intramuscular injection. To cannulate the liver, the portal vein was dissected to the superior border of the pancreas. The livers were perfused with perfusate (NaCl 8.3 g/L, KCl 0.5 g/L, EGTA 0.95 g/L, and HEPES 2.4 g/L) to remove the blood. The organs were then rinsed twice with phosphate-buffered saline (PBS) and stored at −20 °C until use.

### Decellularization

The livers were thawed at room temperature. A feeding tube was inserted into the portal vein and connected to a pump for perfusion with solutions. The first solution used was deionizedwater administered at a rate of 200 mL/min for 1 hour. The liver was then perfused with 1% Triton X-100 (Amresco, Solon, OH, USA) at a rate of 200 mL/min for 3 h and then with 1% SDS (Promega, San Luis Obispo, CA, USA) at the same rate for 6 h. This was followed by 3 h of perfusion with 1% Triton X-100 to remove residual SDS. Subsequently, the liver was washed with 20 L of distilled water to remove residual detergent, followed by infusion of 40 L of PBS at 200 mL/min.

### Histological detection of ECM components

To generate liver scaffolds, native livers and decellularized matrix were sliced into square transverse sections (15 mm × 12 mm × 3 mm).The samples used for histology and immunohistochemistry were fixed in 10% buffered formalin for 24 h. Paraffin sections (4 μm thick) were prepared from embedded samples and stained with H&E to visualize to the cellular components and ECM. Nuclear-specific 4,6-diamidino-2-phenylindole (DAPI) staining was performed to assess the degree of nucleus removal. Slides were also stained with Sirius Red (blue/red for proteoglycans/collagens, respectively) according to established protocols. Immunohistochemistry was performed with an antibody against alpha-galactosyltransferase (α-Gal; 1:50; Abcam, Cambridge, MA), swine leukocyte antigen DR (SLA-DRα; 1:50; AbD Serotec, Oxford, UK), and swine leukocyte antigen 2 (SLA2; 1:30; Genetex, San Antonio, TX) to determine the clearance of the Gal epitope, SLA-DRα antigen and SLA2 antigen, respectively. Photomicrographs were taken with an Olympus upright microscope (Olympus, Tokyo, Japan) and an Olympus soft image viewer.

### DNA quantification

The dry weight of the native and decellularized tissues was measured, and DNA was isolated from 30 mg samples using the DNeasy Tissue Kit (Tiangen Biotech, Beijing, China, n = 12/each porcine liver). Total DNA extracts were loaded onto a 2% agarose gel for electrophoresis with a DNA ladder to assess the DNA fragments. Polymerase chain reaction (PCR) examinations were performed based on previously described protocols^44^. The sequences of primers are listed in Table 1.

### Crosslinking

The 0.625% glutaraldehyde (Kelong, Chengdu, China) or 0.625% genipin (Sigma Chemical Co., St. Louis, MO) solution was perfused into the decellularized porcine whole liver through the portal vein andrecycledat 100 mL/min for 12 h, followed by a 12-hour rinsing step in deionized water perfusion, a 12-hour rinsing step in recycled deionized water perfusion and further perfusion in PBS for 6 h at 100 mL/min. Crosslinked decellularized livers were sliced into square transverse sections (5 mm × 12 mm × 3 mm). All groups of liver materials were placed in 24-well plates, vacuum dehydrated, sterilized via 1 Mrad (10 kGy) γ irradiation, and then stored at 4 °C until use.

### Assessment of modified ECM

Liver materials used for light microscopy were stained with H&E for general histological evaluation. Electron micrographs of sample cross-sections were obtained at 5.0 kV and 500× magnification using a Hitachi S-4800 scanning electron microscope (Tokyo, Japan). GAGs were quantified using the Blyscan GAG assay kit (Biocolor, Belfast, UK) following the manufacturer’s instructions. The collagen content was quantified using a colorimetric assay to detect hydroxyproline following a modification of Grant’s method^45^. Immunohistochemistry was performed to determine the retention of the important basement membrane proteins as well as important growth factors after the crosslinking process. The primary antibodies against collagen-I (1:1,000; GeneTex, Irvine, CA), fibronectin (1:50; Santa Cruz Biotechnology, Santa Cruz, CA), HGF (1:30; Santa Cruz Biotechnology, Santa Cruz, CA), VEGF (1:50; Santa Cruz Biotechnology, Santa Cruz, CA), bFGF (1:50; Santa Cruz Biotechnology, Santa Cruz, CA) and IGF (1:50; Santa Cruz Biotechnology, Santa Cruz, CA) were used. Quantification was performed in at least 10 random views on each slide using Image Pro-plus software (5.0 ver., Media Cybernetics, Silver Spring, MD).

### Cell seeding on liver decellularized scaffolds

Decellularized ECM from the decellularized, genipin and glutaraldehyde groups were equilibrated in fetal bovine serum (FBS) for 12 h at 4 °C and then soaked in DMEM culture medium (for the EA.hy926 culture) or serum-free medium (for the hepatocyte culture).Wells containing the same amount of cells without scaffold were used as the control group. The effect of ECMs on endothelial cell proliferation was evaluated using human EA.hy926 cells. The EA.hy926 cells were inoculated into 6-well plates along with the ECM materials (n = 6/each group) at a density of 1 × 10^6^ cells/cm^2^ and then cultured in DMEM medium supplemented with 10% FBS. Primary rat hepatocytes were also used to evaluate the biocompatibility of liver crosslinked matrices. Hepatocytes were isolated from a 200 g male Sprague-Dawley rat by a two-step perfusion protocol, as we previously described^46^. After isolation, the hepatocytes were cultured with ECM materials (n = 6/each group) in a 6-well plate at a density of 1 × 10^6^ cells/cm^2^ in our serum-free medium. Cell viability was determined 3 days after cell seeding using a Cell Counting Kit-8 (Dojindo Laboratories, Kumamoto, Japan). Briefly, CCK-8 solution (culture medium: CCK-8 solution = 10:1) was added to each well and the plate was incubated in a humidified 5% CO_2_ atmosphere for 3 hours at 37 °C. Then, 100 μL of the medium in each well was transferred to a 96-well plate, and the absorbance at 450 nm was measured using a microplate reader. The viable cell samples cultured on scaffolds were examined by H&E staining.

### Hepatocyte function assays

Culture medium was also collected at day 3, day 5 and day 7 for hepatocyte functional evaluation. Hepatocytes were also cultured in a sandwich configuration as a 3D control, the seeding density and media was the same as decellularized scaffolds culture. Briefly, ice-cold 0.25 mg/ml Matrigel^TM^ (BD Biosciences, Heidelberg, Germany) diluted in incubation media at 1.5 ml/well were added to the 6-well plates and then incubated at 37 °C for 2 h. Excess Matrigel^TM^ was removed by aspirating media and rinsing with PBS. Hepatocytes were then added onto the Matrigel^TM^-coated bottom of the plates and allowed seeding for 24 h at 37 °C. Then the media was aspirated and replaced with another 1.5 ml/well Matrigel^TM^ solution, and the plates were incubated for 2 h before media change for the upper collagen layer to be fixed. The normal single layer culture was used as a 2D control. The media was replaced every day and the culture ended at day 7. Albumin was measured with a rat albumin ELISA quantification kit from Bethyl Laboratories (Montgomery, TX) according to the manufacturer’s instructions. Measurements of urea were performed using a QuantiChrom^TM^ DIUR-500 Urea Assay Kit (BioAssay System, Hayward, CA). All assays were performed in triplicate.

### Liver material implantation

Sprague-Dawley (n = 36) rats weighing approximately 300 g were obtained from the animal center at West China Hospital, Sichuan University. Rats were kept in a temperature-controlled room with a 12 h/12 h light/dark cycle control and provided with an adequate rodent diet and water. All rats were randomly assigned to 4 groups (n = 9 for each group) based on sample type. A partial-thickness abdominal wall defect model^16^ was used in the present study to assess the rat host immune responses to xenogenic materials. Rats were anesthetized by intraperitoneal administration of sodium pentobarbital (25 mg/kg) and placed on a warm plate. A ventral midline abdominal skin incision was created, and the skin and subcutaneous tissue were then separated from the muscle on the right side of the midline to the anterior axillary line. A square section (side length = 1 cm) of the external and internal oblique muscles on the ventral lateral abdominal wall was created. The defect was repaired with a prepared sample of the chosen material. The skin was closed, and animals were returned to their cages with no immunosuppressive agents administered. Animals were sacrificed at 7, 14 and 21 days post-implantation (n = 3/time point/group), and both implants and surrounding tissues were collected for further assessment.

### Histology and immunofluorescence

H&E staining was conducted to observe the grafts for signs of immunological rejection. All slides were evaluated histologically by a proficient pathologist under a microscope in a blinded fashion. For immune cell quantification, neutrophils are round, 10–12 μmin diameter, with a lobe-like or polymorphous nucleus, whereas lymphocytes are 5–10 μm spot-like cells that have a round nucleus with indentations and little cytoplasm. The infiltrated immune cells were counted in at least five different areas for each slide at 200× magnification by the same pathologist. For vascular cells and fibroblast immunochemistry, anti-CD31 antibodies and anti-vimentin antibodies were used. For macrophage phenotype assessment, immunofluorescent co-staining was performed with primary antibodies of CCR7 (1:100, abcam, Cambridge, MA), CD206 (1:75; Santa Cruz Biotechnology, Santa Cruz, CA) and CD68 (1:100, abcam, Cambridge, MA). CCR7 is an M1 macrophage marker, CD206 is an M2 macrophage marker, and CD68 is a pan-macrophage marker. DAPI staining was used as a nuclear counterstain. All the slides were observed under a Leica Microsystems DM40000B upright microscope (Leica Microsystems, Inc., Wetzlar, Germany) with appropriate fluorescent filter sets and bright field.

### Isolation of PBMCs

All the protocols involving human blood were approved by the Ethics Committee of West China Hospital, Sichuan University. The experiments were performed in accordance with the approved guidelines of the Ethics Committee of West China Hospital, Sichuan University. Human leukocytes were isolated from healthy volunteers (n = 6 males, n = 2 females; mean age of 29.8 ± 5.0) who had signed informed consent forms. Blood samples were added to lymphocyte separation medium (MP Biomedicals, Solon, OH). Human PBMCs were aspirated from the buffy coat following standard density centrifugation at 400 × g and 25 °C for 20 minutes. The interface PBMCs were washed three times with PBS containing a 10% v/v penicillin/streptomycinmixture at 300× g and 25 °C for 10 minutes, and the cells were then counted using a Fuchs Rosenthal cell chamber.

### Preparation of tissue extracts

Samples from the four groups were weighed and ground with a mortar and pestle in 2 mL of ice-cold RPMI 1640 medium. The homogenates were centrifuged at 12,000× g and 18 °C for 30 minutes. The supernatants were carefully withdrawn and transferred into new centrifuge tubes. Protein content quantification was performed using a NanoDrop spectrophotometer (ND-2000c; Thermo, Waltham, MA). All of the protein extracts were diluted with RPMI 1640 medium to a protein concentration of 1 mg/mL. Sterile technique was carried out throughout the experiments.

### Preparation of OKT3-coated microtiter plates

The anti-CD3 monoclonal antibody OKT3 (Novartis Pharma AG, Basel, Switzerland) was used at a suboptimal dose to stimulate human leukocyte proliferation in a CFSE-based proliferation assay according to a previously described protocol^47^. This dose was reported to not induce T cells proliferation or at merely low proliferation rates, and its efficiency in this research was further confirmed. Briefly, OKT3 was dissolved in PBS to a final concentration of 0.1 mg/mL. Exactly 50 μL of antibody solution was added to each well of a flat-bottom 96-well culture plate (BD Falcon, Newton, MA). The 96-well plate was placed at 4 °C for 20 hours. After the incubation period, the wells were washed three times with PBS containing a 10% v/v penicillin/streptomycinmixture.

### CFSE staining and assay of immune cell subpopulation proliferation

Isolated PBMCs were subsequently resuspended in PBS to a final concentration of 10^7^ cells/mL for CFSE labeling. CFSE was added to the cell suspension to a final CFSE concentration of 5 mM and incubated at room temperature for 5 minutes. The staining was stopped by adding ten volumes of ice-cold PBS containing 10% v/v fetal bovine serum (FBS). Finally, the labeled cells were resuspended in RPMI 1640 medium supplemented with 20% v/v inactivated autologous human serum. A total of 100 μL of RPMI 1640 medium containing 3 × 10^5^ CFSE-labeled cells was added to each well of the 96-well plate, along with 100 μL of RPMI 1640 medium supplemented with protein extract. Aliquots of 200 μL of medium containing 3 × 10^5^ cells in OKT3-coated and non-coated wells served as negative controls. Concanavalin A (ConA, Sigma, Steinheim, Germany) at a concentration of 5 mg/mL served as a positive control due to its strong ability to induce immune cell proliferation. For each time point of PBMC isolation, 3 wells were used for each group. All of the wells were incubated in a humidified 5% CO_2_ atmosphere for 5 days at 37 °C. After incubation, the co-cultures were centrifuged at 300× g and 25 °C for 10 minutes, and the supernatants were collected and frozen at −80 °C for further analysis. Mouse anti-human CD3-APC, CD4-PerCP-Cy5.5, CD8-PE, CD19-APC, or HLA-DR-PE (BioLegend, San Diego, CA) or the corresponding mouse isotype-control antibodyIgG2a-PE, IgG1-APC, IgG1-PerCP-Cy5.5, or IgG2a-APC (BioLegend, San Diego, CA) was used to label the PBMCs. The CFSE intensity was measured by flow cytometry using a Navios Flow Cytometer (Beckman Coulter, Fullerton, CA).

### Enzyme-linked immunosorbent assay (ELISA)

Supernatants of the PBMC and protein extract co-cultures were analyzed to identify the levels of IgG and IgM secretion. A human IgG ELISA kit (Abcam, Cambridge, MA) and human IgM ELISA kit (Abcam, Cambridge, MA) were used following the manufacturer’s instruments. The absorbance was measured in a Sunrise^TM^ microplate reader (TECAN, Mannedorf, Switzerland). Allassays were performed in triplicate.

### Th1/Th2 cytokine detection

Both the supernatants of the PBMC cultures in the CFSE-based proliferation assay and the protein extracts fromrat abdominal wall grafts were analyzed for their Th1/Th2 cytokine profile. For protein extracts, the graft tissues were lysed using RIPA lysis buffer (moderate; Beyotime, Shanghai, China), and the protein levels were equalized to a concentration of 5 mg/mL. Th1/Th2 cytokine profiles were determined using a Luminex^®^ xMAP^TM^ Kit (Millipore, Billerica, MA) following the manufacturer’s instructions. This methodology utilized microbeads coated with purified Th1 or Th2 cytokine antigens and included pre-optimized reagents for the analysis of the Th1/Th2 balance. The levels of IL-2, IL-4, IL-5, IL-6, IL-10, TNF-α, and IFN- γ in four groups were assessed. PBS containing 0.1% Triton X-100 and 5 mg/mL bovine serum albumin was used as a control. At least 100 beads were counted per analyte. All assays were performed in triplicate.

### Statistical analysis

All values are shown as the mean ± standard error of the mean (SEM). To determine significant differences between individual groups, the results were analyzed using analysis of variance (ANOVA). A p-value of less than 0.05 was considered statistically significant. At least 3 parallel experiments were conducted using different samples. All data were organized using GraphPad Prism software (La Jolla, CA), and statistical analyses were performed using SPSS 19.0 (Chicago, IL).

## Additional Information

**How to cite this article**: Wang, Y. *et al.* Genipin crosslinking reduced the immunogenicity of xenogeneic decellularized porcine whole-liver matrices through regulation of immune cell proliferation and polarization. *Sci. Rep.* **6**, 24779; doi: 10.1038/srep24779 (2016).

## Supplementary Material

Supplementary Information

## Figures and Tables

**Table 1:** PCR primer sequences and product length.

Gene	Abbr.	Forward primer	Reverse primer	Size of product
Alpha galactosyltransferase	α-gal	5′-GCTCCACCTGGCAGTCATAG-3′	5′-GTCCTGGAGGATTCCCTTGA-3′	361 bp
Porcine endogenous retrovirus	PERV	5′-CTACCCCGAGATTGAGGAGC-3′	5′-GGGGGATGGTTAGTTTTCCA-3′	317 bp
Swine leukocyte antigen DR alpha	SLA-DRα	5′-CGAGAAGAGGTGGCAAGACA-3′	5′-GTCCTGGAGGATTCCCTTGA-3′	220 bp
Swine leukocyte antigen 2	SLA-2	5′-GTCACCTTGAGGTGCTGGG-3′	5′-TGGCAGGTGTAGCTCTGCTC-3′	185 bp
