# Supplementary material for: Genipin crosslinking reduced the immunogenicity of xenogeneic decellularized porcine whole-liver matrices through regulation of immune cell proliferation and polarization
Source: Sci Rep. 2016 Apr 21;6:24779. doi: 10.1038/srep24779 (PMC4838870; doi:10.1038/srep24779)
Supplement: Supplementary Information [file srep24779-s1.pdf]

Genipin crosslinking reduced the immunogenicity of xenogeneic decellularized porcine whole-liver matrices through regulation of immune cell proliferation and polarization

Yujia Wang[1,3,5], Ji Bao[1,3,5], Xiujuan Wu[4], Qiong Wu[1,3], Yi Li[1,3], Yongjie Zhou[1,3], Li Li[1,3], Hong Bu[1,2,3]*

1. Laboratory of Pathology, West China Hospital, Sichuan University, Chengdu, 610041, China

2. Department of Pathology, West China Hospital, Sichuan University, Chengdu, 610041, China

3. Key Laboratory of Transplant Engineering and Immunology, Ministry of Health, West China Hospital, Sichuan University, Chengdu, 610041, China

4. Department of General Surgery, The first people's hospital of Yibin, Yibin, 644000, China

5. These authors provided equal contribution to this work.

* Corresponding author. Tel: +86-28-85164030; Fax: +86-28-85164033. E-mail address: hongbu@scu.edu.cn (H. Bu)

Supplementary Information

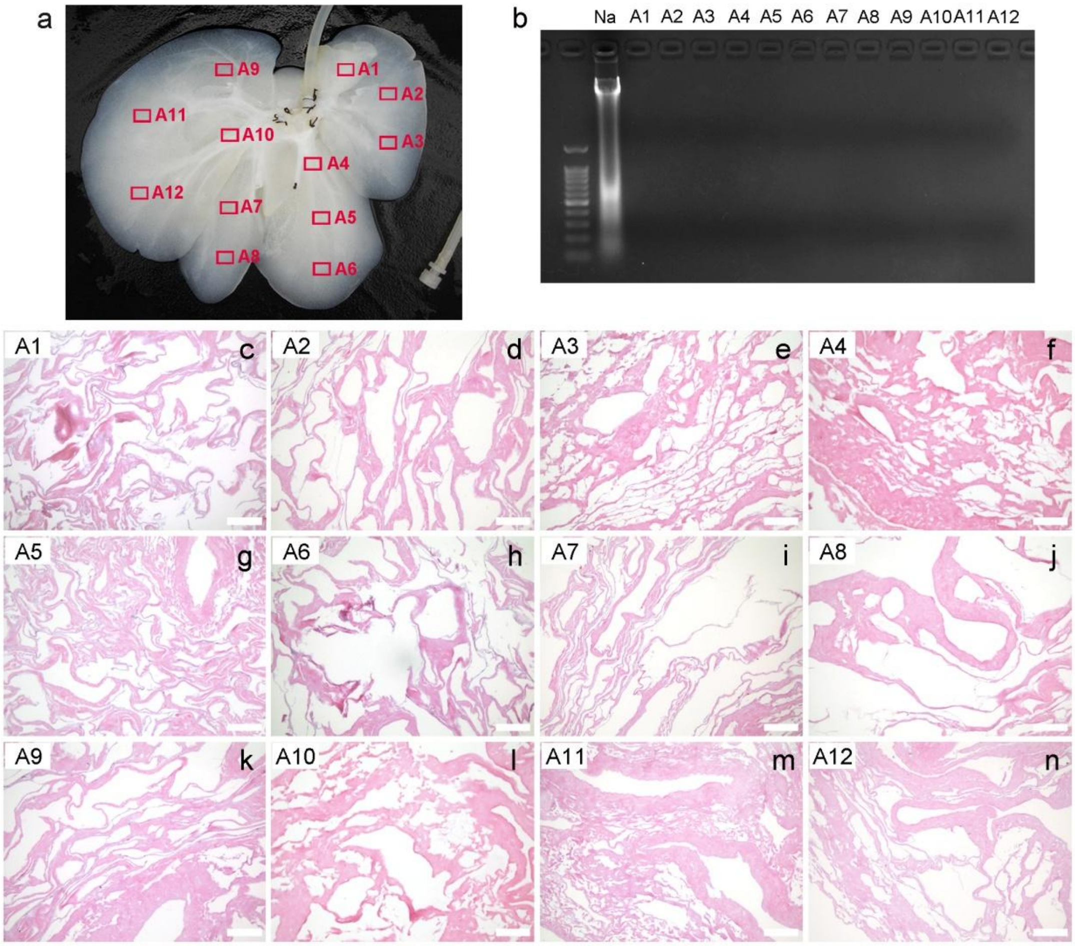

Supplementary Fig. S1 Analysis of decellularization uniformity.

(a) The morphology of decellularized liver and the indicated 12 different areas. (b) Agarose gel electrophoresis of DNA extracted from selected decellularized liver matices. Na=native control. (c-n) Histological images of selected liver ECM stained with H&E indicating decellularization uniformity. Scale bars = 100 μm.

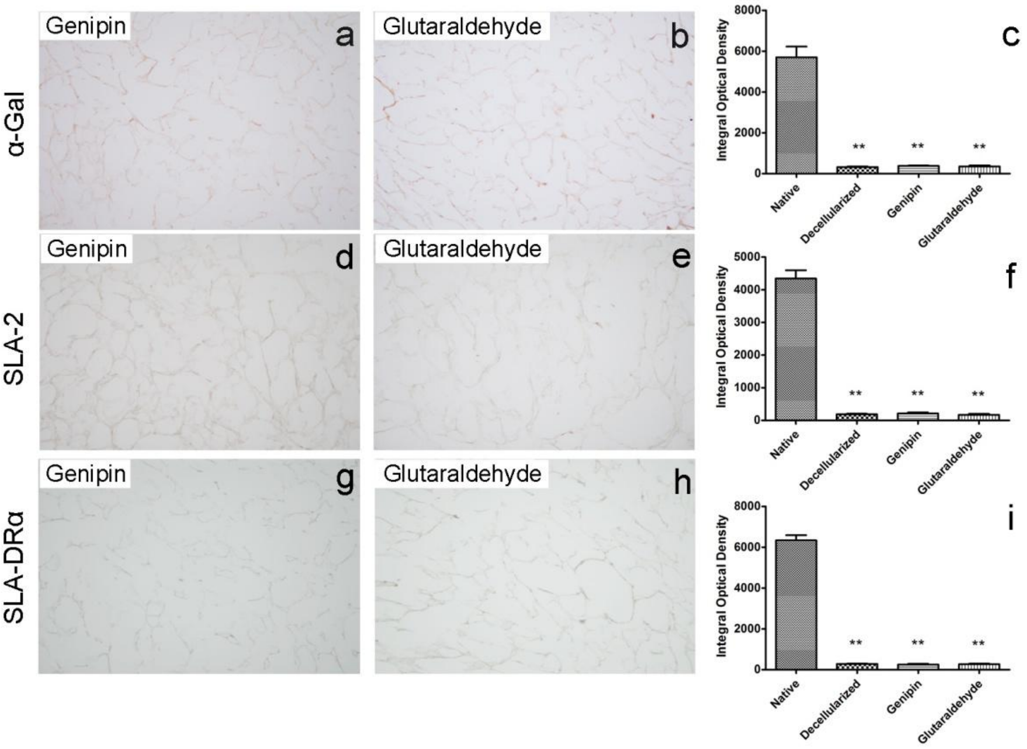

Supplementary Fig. S2 Immunohistochemistry and quantification of immunogenic or pathogenic antigens on matrix.

Immunohistochemical staining of genipin and glutaraldehyde crosslinked liver ECMs for α-Gal (a, b), SLA-2 (d, e), SLA-DRα (g, h) are shown. Scale bars = 100 μm. Immunohistochemical quantification ofα-Gal (c), SLA-2 (f), SLA-DRα (i) are also shown. $^{**}p<0.01$ with respect to the native group. All data are given as the mean ± SEM.

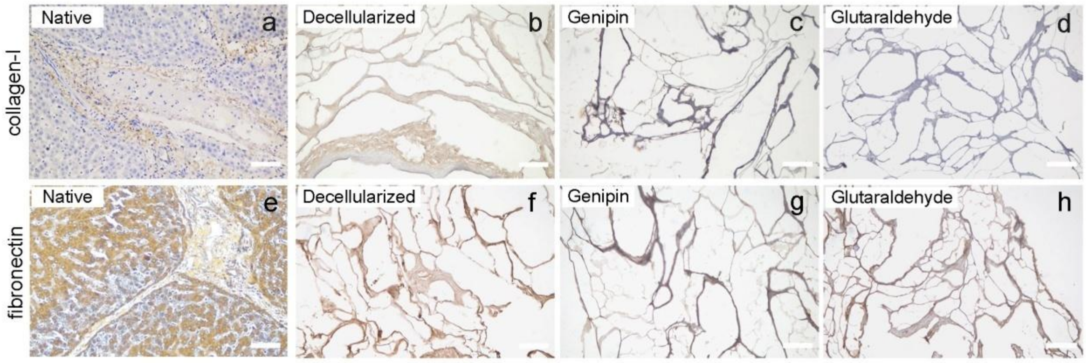

Supplementary Fig. S3 Immunohistochemistry of matrix components.

(a–p) Immunohistochemistry staining of native liver and un-crosslinked and crosslinked decellularized liver ECM for collagen I and fibronectin. Scale bars = 100 μm.

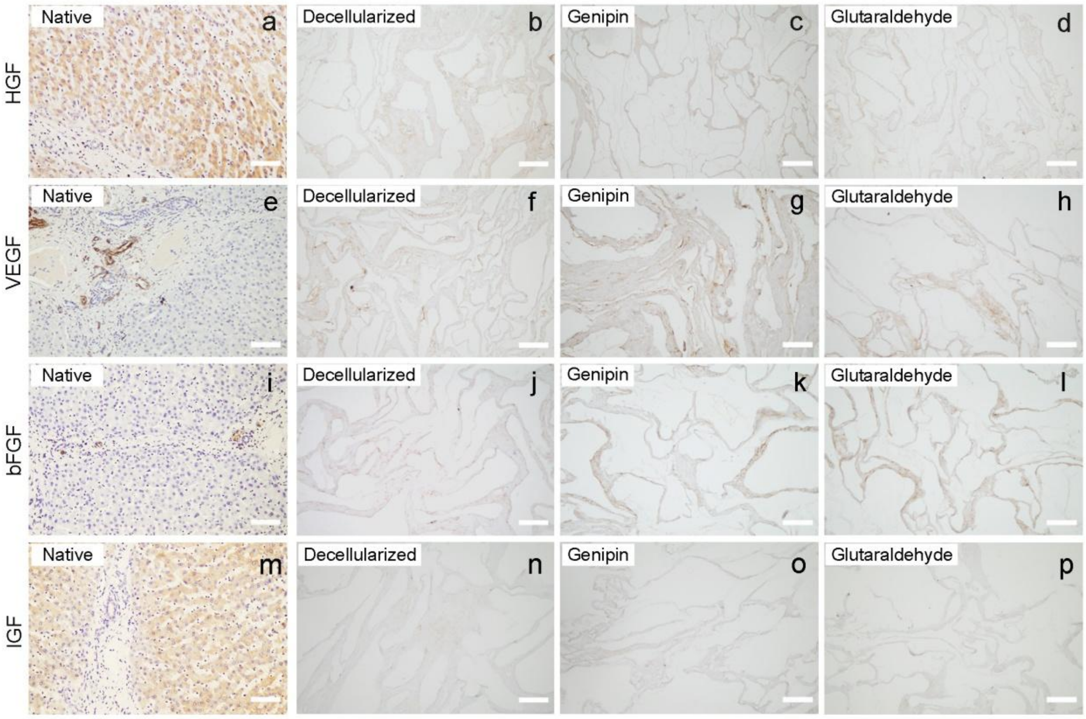

Supplementary Fig. S4 Immunohistochemistry of residual growth factors in liver matrix.

(a–p) Immunohistochemistry staining of native liver, un-crosslinked and crosslinked liver ECM for HGF, VEGF, bFGF, and IGF (from top to bottom). Scale bars = 100 μm.

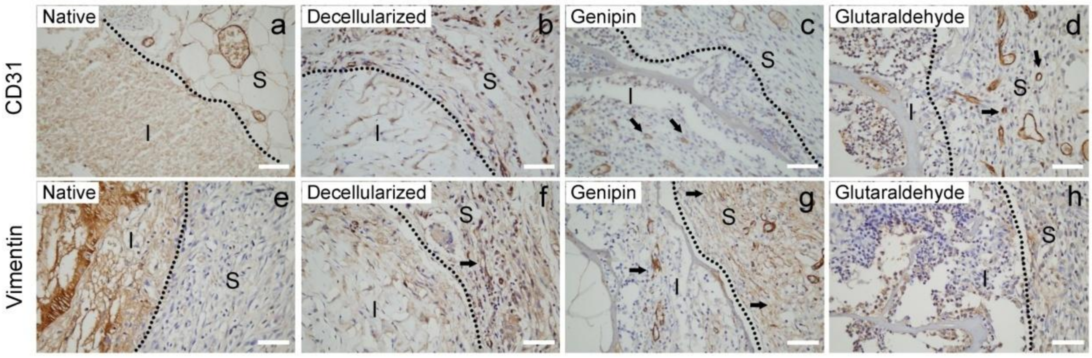

Supplementary Fig. S5 Immunochemistry of the liver xenografts.

(a-h) Immunochemistry towards CD31 and vimentin were examined in native liver, decellularized liver ECM, genipin-fixed ECM, and glutaraldehyde-fixed ECM at 21 days post-surgery to show the vascular endothelial cells (arrowed) and fibroblast cells (arrowed). Scale bars = 50 μm. The dotted line indicates the border of the implants and surrounding tissue. Abbreviations: S=surrounding tissue; I=implanted porcine liver materials.

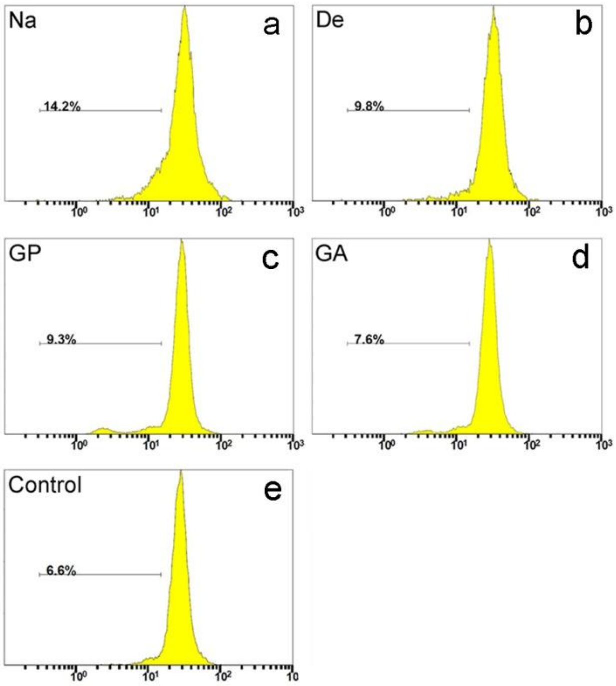

Supplementary Fig. S6 Proliferation properties of PBMCs in co-culture with liver matrices without OKT3.

(a-e) Representative FACS histograms of immune cells co-cultured without OKT3 stimulus.

Proliferation responses without protein extracts were used as a negative control.

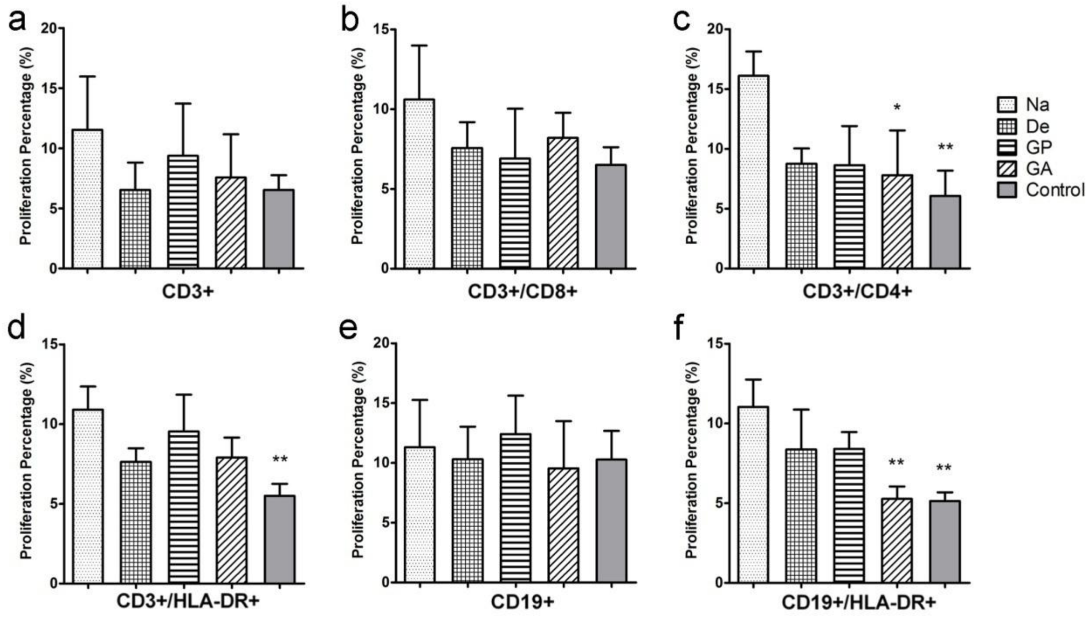

Supplementary Fig. S7 Impact of porcine liver matrices on T cell and B cell subpopulation proliferation in co-cultures without CD3 stimulus.

(a-f) The proliferation patterns of T cells, B cells and their subsets was analyzed using anti-human CD3, CD8, CD4, HLA-DR, CD19 antibodies. *$p<0.05$ with respect to Na group, **$p<0.01$ with respect to Na group. All data are given as the mean ± SEM (n=3).

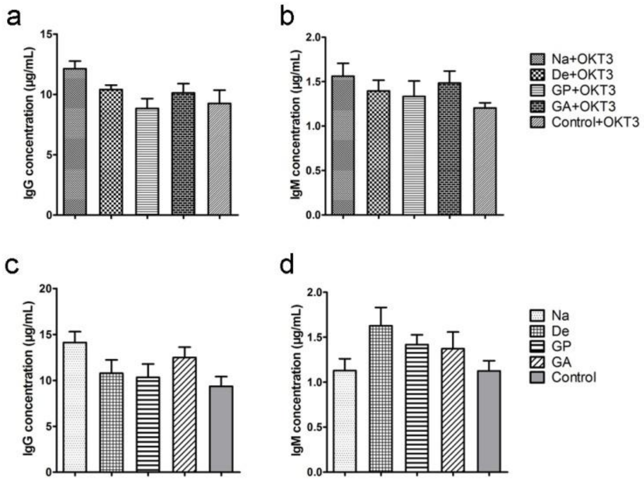

Supplementary Fig. S8 Impact of porcine liver matrices on B cell activation *in vitro*.

IgG and IgM production in human PBMC co-culture with liver material extracts in the presence of OKT3 (a,b) or without OKT3 (c,d).

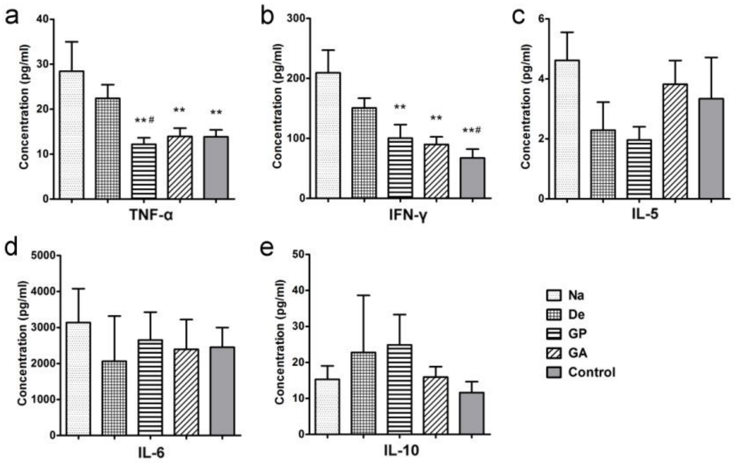

Supplementary Fig. S9 Th1/Th2 cytokine secretion profile of PBMCs co-cultured with protein extracts and without anti-CD3 stimulus.

(a-e) Th1/Th2 cytokine levels are shown for co-cultures of PBMCs alone or in combination with protein extracts of porcine matrix. **p<0.01 with respect to native group, #p<0.05 with respect to De group. All data are given as the mean ± SEM (n=3).

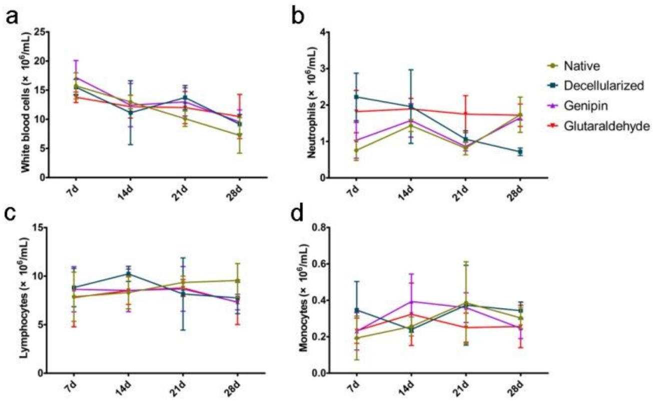

Supplementary Figure. S10 Systemic white blood cell counts of host rats.

(a-d) total white blood cell count, neutrophil count, lymphocyte count, and monocyte count are shown, indicating no differences among groups. All data are given as the mean ± SEM (n=3).
